# Effect of glycotoxicity and lipotoxicity on carbohydrate antigen 19 − 9 in the patients with diabetes

**DOI:** 10.1186/s12902-024-01578-5

**Published:** 2024-04-24

**Authors:** Xi-yu Liu, Xiao-hong Wang

**Affiliations:** The Affiliated Dongyang Hospital of Wenzhou Medical University, Dongyang, Zhejiang China

**Keywords:** Diagnosis, Diabetes, Tumor marker, Carbohydrate antigen 19-9 CA 19-9 diabetes, Glycotoxicity, Hyperglycemia

## Abstract

**Objectives:**

In comparison to the subjects without diabetes, a greater concentration of serum carbohydrate antigen 19 − 9 (CA 19 − 9) was observed in the subjects with diabetes. Nevertheless, since the occurrence of abnormal CA 19 − 9 is not widespread among the whole diabetic population, this phenomenon has not attracted enough attention. The prevalence of abnormal CA 19 − 9 in hospitalized patients with diabetes was the focus of our research.

**Method:**

A total of 385 subjects with diabetes and 200 controls were enrolled and all had been tested the CA19-9 levels. Cases of cancers were excluded through examination and followup for 1 year.

**Results:**

We found that the rate of patients with abnormal CA19-9 level was 8.3%. The rate of patients with abnormal CA19-9 level was 14.0% in the HbA1c ≥ 9% group, and 3.0% in the HbA1c < 9% group, 2.5% in the control group. There was no significant difference in the HbA1c < 9% group and the control group. A significant correlation between serum CA19-9 and both HbA1c and total cholesterol was observed, yet no difference in CRP level was observed between subjects with normal CA19-9 level and subjects with abnormal CA19-9 level. However, a significant difference in fasting C-peptide levels was observed between the two groups, *p* = 0.039.

**Conclusion:**

The percentage of patients with diabetes exhibiting elevated CA19-9 level is 14% in the HbA1c ≥ 9% diabetic patients, much higher than expected. The underlying mechanism may be related to islet injury caused by glycotoxicity and lipotoxicity.

**Strengths and limitations of the study:**

We studied the rate of hospitalized diabetic patients with elevated CA 19 − 9 which were characterized with poorly controlled blood glucose. We found that the elevation of CA 19 − 9 was unexpectedly high in diabetic inpatients without development to cancer. The limitation of this study is that the underlying mechanism is not sufficiently studied.

## Introduction

The marker of Carbohydrate antigen 19 − 9 (CA 19 − 9) is widely used for screening and monitoring of cancers [1, 2]. However, data from asymptomatic subjects were used to analyze the diagnostic performance of CA 19 − 9 for cancers [3]. It showed that for gastrointestinal cancers, the sensitivity and positive predictive value of CA19-9 were 7.4% and 2.7%, and for cancers (including other organ cancers), the sensitivity and positive predictive value of CA19-9 were 10.8% and 5.8%. It suggested that there are other non-malignant conditions that could cause the elevation of CA19-9. Some research showed that benign biliary conditions, metabolic diseases, inflammatory diseases could cause the elevation of CA 19 − 9 [4–6]. Kim S [7] studied the causes of CA 19 − 9 elevation without evidence of malignant or pancreatobiliary diseases. They found that hepatic diseases, pulmonary diseases, gynecologic diseases and endocrine diseases could cause the elevation of CA 19 − 9. Another study found that the patients with diabetes had higher levels of CA19-9 level than the subjects without diabetes (the CA19-9 level was 13.0 versus 7.25 U/ml, *p* < 0.001), and the CA19-9 levels were associated with hyperglycemia [8]. However, these studies just compared the CA 19 − 9 level between the subjects with diabetes and those without diabetes. The prevalence of abnormal CAn19-9 among hospitalized diabetic patients with a large proportion of poor glycemic control was not reported. Our study focused on the hospitalized diabetic patients with a large proportion of poor glycemic control (HbA1c ≥ 9%). We found a unexpectedly high percentage of abnormal CA 19 − 9 in diabetic patients with poor glycemic control.

## Methods

### Population

Our study was in compliance with the Helsinki Declaration. The ethics approval was issued by the Ethics Committee. All researchers promised to keep patient information confidential. The informed consent was obtained from all subjects. Procedure for enrolling patients was as follows. A total of 604 hospitalized patients with diabetes was screened from our hospital between January 1, 2019 and July 1, 2019. Among them, 436 patients had complete case information, including diagnosis, metabolic index and were checked the level of CA 19 − 9. Thirty-one patients had tumor after examination or followup and were excluded. Nineteen patients were excluded because of severe liver or kidney diseases, and one pregnant patient was excluded. Ultimately, A total of 385 diabetic patients were included in this study for statistical analysis.

And 200 controls without diabetes who were matched for sex and age to the diabetes group were enrolled.

### Patient and public involvement

Patients or the public were not involved in the design, or conduct, or reporting, or dissemination plans of our research.

### Inclusion criteria and exclusion criteria

The inclusion criteria of diabetic patients group were all of the following: (1) Hospitalized diabetic patients with complete medical information. (2) The CA 19 − 9 level was detected. (3) Follow up for more than 1 year.

The exclusion criteria of diabetic patients group were as follows: (1) A history of tumor. (2) Pregnant or lactating women. (3) Insufficiency of kidney function with estimated glomerular filtration rate < 60 ml/min/1.73 m^2^. (4) Liver cirrhosis or liver insufficiency with alanine aminotransferase 3 times higher than the upper limit of normal range. (5) Malignant tumor was found during examination or follow-up.

The inclusion criteria of controls were as follows: (1)Individuals with no history of diabetes (2) The CA 19 − 9 level was detected.

The exclusion criteria of controls were as follows: (1) A history of tumor. (2) Pregnant or lactating women. (3) Insufficiency of kidney function with estimated glomerular filtration rate < 60 ml/min/1.73 m2. (4) Liver cirrhosis or liver insufficiency with alanine aminotransferase 3 times higher than the upper limit of normal range. (5) Malignant tumor was found during examination. (6) Abnormal HBA1c or fasting blood glucose was found through the physical examination.

### Detection method

Blood samples for detection were collected in the morning after an overnight fast. CA19-9 and C peptide were measured by an electrochemiluminescence immunoassay [9], according to the manufacturer’s instructions (Roche Diagnostics, Germany). Glycohemoglobin (HbA1c) was detected by high-performance liquid chromatography [10] (Bio-Rad Laboratories, France). Blood glucose, cholesterol, triglyceride, uric acid, liver function and kidney function were measured using Colorimetric/Fluorometricassay (Fujifilm Wako Pure Chemical Corporation, Japan).

### Statistical analysis

IBM SPSS Statistics 25.0 was used for statistical analysis. Independent sample t test was used for comparison of continuous variables. Kruskal-Wallis was performed before independent sample t test between the groups. The rates between two groups were compared by Chi-square test. Binary logistic regression analysis was used to explore the risk factors of CA19-9 level.

## Results

### The different rate of patients with abnormal CA19-9 between different glycemic control status

**Fig. 1:**
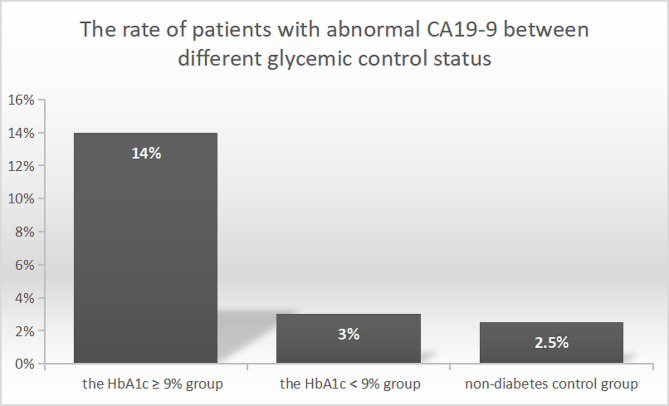
The rate of subjects with abnormal CA19-9 level in different glycemic status group 1, the HbA1c ≥ 9% diabetes group (*n* = 186); group 2, the HbA1c < 9% diabetes group (*n* = 199); group 3, the non-diabetes control group (*n* = 200). There was a significant difference between group 1 and group 2, group 1 and group 3, *p* value was less than 0.01. There was no difference between group 2 and group 3

A total of 385 hospitalized patients with diabetes and 200 controls without diabetes were included in the study for statistical analysis. The percentage of subjects with diabetes exhibiting elevated CA 19 − 9 level (CA19-9 level ≥ 39U/ml) was 8.3% (32/385). We divided the patients with diabetes into two groups to investigate whether CA 19 − 9 levels differed under different glycemic control status, including the HbA1c < 9% group, and the HbA1c ≥ 9% group with poorly glycemic control. Basic demographic and clinical characteristics of diabetic patients were showed in Table 1. The percentage of subjects with diabetes exhibiting elevated CA 19 − 9 level was significantly different, 14.0% in the HbA1c ≥ 9% group, and 3.0% in the HbA1c < 9% group, *p* < 0.001. It was shown in Fig. 1.

**Table 1:** Basic demographic, clinical characteristics and CA 19 − 9 levels of diabetic inpatients

	The HbA1c < 9% group (*n* = 199)	The HbA1c ≥ 9% group (*n* = 186)	*p* value
CA 19−9 levels (U/ml)	14.8 ± 9.6	22.0 ± 17.4	< 0.001*
Proportion of patients with abnormal CA 19−9	6/199 (3.0%)	26/186 (14.0%)	< 0.001*
Age (years)	59.9 ± 11.6	55.4 ± 13.3	< 0.001*
Female sex	62 (31.2%)	49 (26.3%)	0.298
BMI (kg/m2)	25.2 ± 3.2	24.8 ± 3.7	0.240
HbA1c (%)	7.26 ± 0.98	11.37 ± 1.91	< 0.001*
Systolic blood pressure (mmHg)	133.4 ± 19.7	128.9 ± 20.2	0.027
Diastolic blood pressure (mmHg)	79.9 ± 11.5	81.5 ± 12.1	0.173
Uric acid (µmol/l)	330.1 ± 93.8	305.7 ± 94.2	0.012
Total cholesterol (mmol/l)	4.59 ± 1.24	4.77 ± 1.27	0.157
Triglycerides (mmol/l)	1.84 ± 1.64	2.02 ± 1.82	0.314
Creatinine (µmol/l)	72.0 ± 27.6	64.6 ± 23.2	0.005*
ALT (U/L)	25.2 ± 15.0	26.6 ± 17.6	0.406

CA 19 − 9, carbohydrate antigen 19 − 9; BMI, body mass index; HbA1c, glycosylated hemoglobin; ALT, alanine aminotransferase; * *p* value was less than 0.01

The clinical characteristics and CA 19 − 9 level of the HbA1c < 9% group and the control group without diabetes were shown in Table 2. The rate of subjects with abnormal CA19-9 level was 2.5% in the control group, and 3.0% in the HbA1c < 9% group, there was no significant difference between the two groups. The clinical characteristics and CA 19 − 9 level of the HbA1c ≥ 9% group and the control group were shown in Table 3. There was no difference in age, sex, blood pressure, urine acid, triglycerides and cholesterol between the two groups. There was a significantly difference in the rate of objects with abnormal CA19-9 level, *p* < 0.001.

**Table 2:** The clinical characteristics and CA 19 − 9 level of the HbA1c < 9% group and control group

Variables	The HbA1c < 9% group (*n* = 199)	Non-diabetes controls (*n* = 200)	*p* value
CA 19−9 levels (U/ml)	14.8 ± 9.6	13.6 ± 8.9	0.128
Proportion of objects with abnormal CA 19−9	6/199 (3.0%)	5/200 (2.5%)	0.753
Age (years)	59.9 ± 11.6	57.5 ± 14.2	0.253
Female sex	62 (31.2%)	64 (32.0%)	0.856
BMI (kg/m2)	25.2 ± 3.2	25.4 ± 3.8	0.422
HbA1c (%)	7.26 ± 0.98	4.8 ± 1.02	< 0.001*
Systolic blood pressure (mmHg)	133.4 ± 19.7	130.1 ± 15.6	0.105
Diastolic blood pressure (mmHg)	79.9 ± 11.5	76.8 ± 11.9	0.182
Uric acid (µmol/l)	330.1 ± 93.8	326.5 ± 91.2	0.243
Total cholesterol (mmol/l)	4.59 ± 1.24	4.68 ± 1.39	0.642
Triglycerides (mmol/l)	1.84 ± 1.64	1.93 ± 1.74	0.506
Creatinine (µmol/l)	72.0 ± 27.6	67.6 ± 15.3	0.081
ALT (U/L)	25.2 ± 15.0	26.9 ± 18.2	0.257

CA 19 − 9, carbohydrate antigen 19 − 9; BMI, body mass index; HbA1c, glycosylated hemoglobin; ALT, alanine aminotransferase; * *p* value was less than 0.01

**Table 3:** The clinical characteristics and CA 19 − 9 level of the HbA1c ≥ 9% group and control group

Variables	The HbA1c ≥ 9% group (*n* = 186)	Non-diabetes controls (*n* = 200)	*p* value
CA 19−9 levels (U/ml)	22.0 ± 17.4	13.6 ± 8.9	< 0.001*
Proportion of objects with abnormal CA 19−9	26/186 (14.0%)	5/200 (2.5%)	< 0.001*
Age (years)	55.4 ± 13.3	57.5 ± 14.2	0.153
Female sex	49 (26.3%)	64 (32.0%)	0.222
BMI (kg/m2)	24.8 ± 3.7	25.4 ± 3.8	0.587
HbA1c (%)	11.37 ± 1.91	4.8 ± 1.02	< 0.001*
Systolic blood pressure (mmHg)	128.9 ± 20.2	130.1 ± 15.6	0.438
Diastolic blood pressure (mmHg)	81.5 ± 12.1	76.8 ± 11.9	0.267
Uric acid (µmol/l)	305.7 ± 94.2	326.5 ± 91.2	0.122
Total cholesterol (mmol/l)	4.77 ± 1.27	4.68 ± 1.39	0.382
Triglycerides (mmol/l)	2.02 ± 1.82	1.93 ± 1.74	0.424
Creatinine (µmol/l)	64.6 ± 23.2	67.6 ± 15.3	0.125
ALT (U/L)	26.6 ± 17.6	26.9 ± 18.2	0.691

CA 19 − 9, carbohydrate antigen 19 − 9; BMI, body mass index; HbA1c, glycosylated hemoglobin; ALT, alanine aminotransferase; * *p* value was less than 0.01

### Logistic regression analysis of CA19-9 in diabetic patients

To explore factors related to the CA19-9 level, we conducted binary logistic regression analysis with CA19-9 level as the dependent variable. We found that the CA19-9 level was significantly correlated to HbA1c and total cholesterol, but it was not correlated to age, sex, smoking or drinking status, BMI, blood pressure, uric acid, triglycerides, serum creatinine, or liver function. It was shown in Table 4.

**Table 4:** Binary logistic regression analysis of CA 19 − 9 levels in diabetic inpatients

Variables	OR	95% confidence interval	*p* value
Age (years)	0.997	0.958–1.038	0.888
BMI (kg/m2)	1.137	0.995–1.301	0.060
HbA1c	1.542	1.294–1.839	< 0.001*
Systolic blood pressure (mmHg)	1.028	0.997–1.061	0.081
Uric acid (µmol/l)	0.998	0.992–1.003	0.400
Total cholesterol (mmol/l)	1.656	1.182–2.321	0.003*
Triglycerides (mmol/l)	0.896	0.681–1.180	0.435
Creatinine (µmol/l)	1.001	0.981–1.021	0.939
ALT (U/L)	1.013	0.990–1.036	0.269
Sex (female)	0.563	0.186–1.708	0.311
Smoking status	0.690	0.244–1.945	0.482
Drinking status	0.831	0.301–2.295	0.722

BMI, body mass index; HbA1c: glycosylated hemoglobin; ALT, alanine aminotransferase;

* *p* value is less than 0.01

### Mechanism study

To investigate the mechanism of elevated CA19-9 in the patients with diabetes, we screened subjects who were tested the CRP level and fasting C-peptide level for further statistical analysis. The subjects with inflammatory diseases were excluded. A total of 309 subjects were included in the mechanism study.

We found that the CRP levels were 2.71 ± 3.65 mg/L in the normal CA19-9 group (*n* = 285), 2.12 ± 1.80 mg/L in the abnormal CA19-9 group (*n* = 24). No significant difference was found in the CRP level between the patients with normal CA19-9 level and those with abnormal CA19-9 level, *p* = 0.438.

**Fig. 2:**
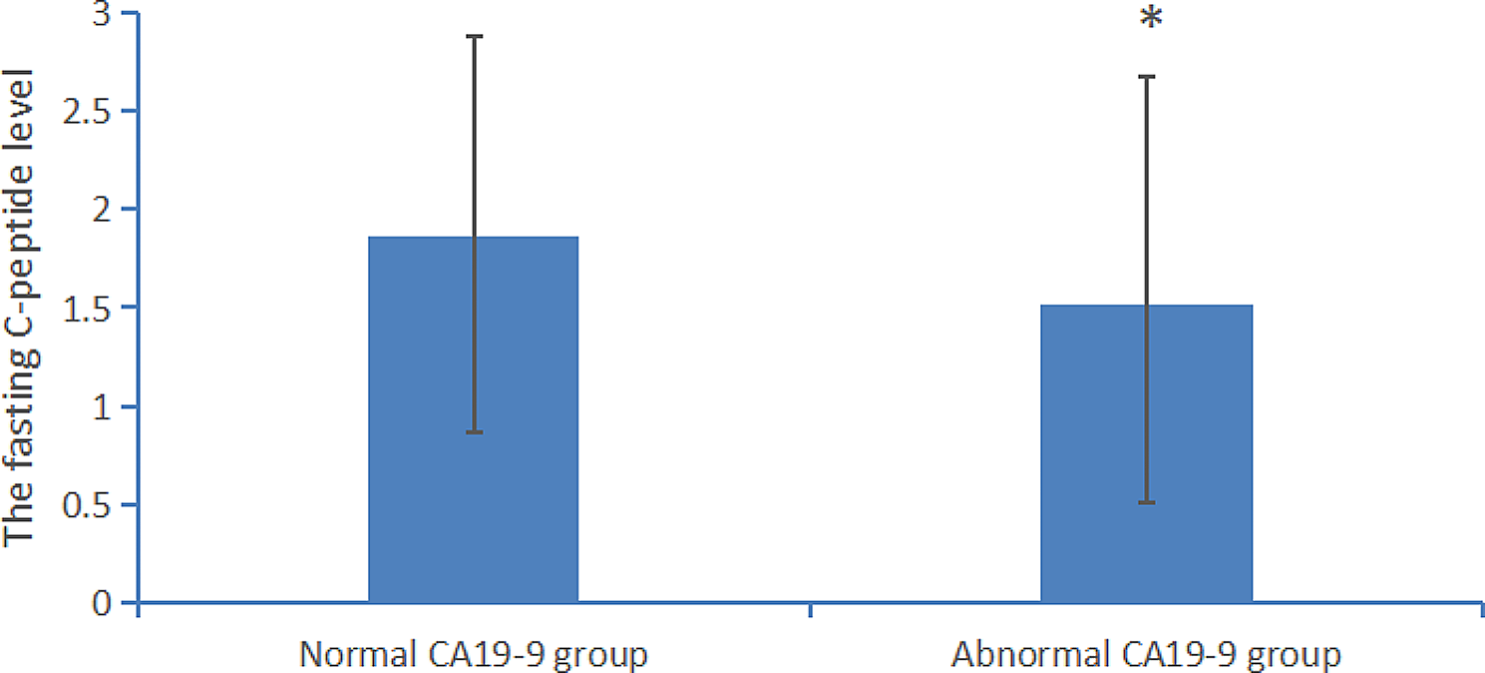
The C peptide level in diabetic patients with normal or abnormal CA 19 − 9 level the normal CA 19 − 9 group (*n* = 360); the abnormal CA 19 − 9 group (*n* = 40). There was a significant difference in the fasting C peptide level between the two groups. *p value was less than 0.05

We found that the fasting C-peptide levels were 1.82 ± 1.00 ng/ml in the normal CA19-9 group (*n* = 285), 1.43 ± 1.07 ng/ml in the abnormal CA19-9 group (*n* = 24), *p* = 0.069. Then, we expanded the number of cases to 400 for further statistical analysis. These patients were still hospitalized patients with diabetes, excluding those with a history of malignancy and those with cancers found during follow-up. We found that the fasting C-peptide levels were 1.86 ± 1.02 ng/ml in the normal CA19-9 group (*n* = 360), 1.51 ± 1.16 ng/ml in the abnormal CA19-9 group (*n* = 40), *p* = 0.039. There was a significant difference in the fasting C-peptide level between the patients with normal CA19-9 level and those with abnormal CA19-9 level, *p* < *0.05* is considered significant. It was shown in Fig. 2.

## Discussion

The marker of CA 19 − 9 is widely used for screening and monitoring the prognosis of cancers, especially pancreatic adenocarcinoma [1, 2, 11]. However, the diagnostic sensitivity and positive predictive value of CA19-9 for cancers is low [3]. The elevation of CA 19 − 9 is also found in several non-malignant diseases, including diabetes [7, 8, 12].

We found a high percentage (14.0%) of abnormal CA 19 − 9 level in diabetic patients with poor glycemic control (HbA1c ≥ 9%). All subjects in our study were excluded malignant tumors by examination or followup. In our study, a significant difference was found in the percentage of subjects with abnormal CA19-9 between the HbA1c ≥ 9% group and the HbA1c < 9% group, *p* < 0.001. Studies have found that the patients with diabetes had higher levels of CA19-9 than the subjects without diabetes [8]. It was conducted to compare the CA 19 − 9 level between the subjects without diabetes and those with diabetes. The participants of our study were all hospitalized diabetic patients with a high proportion of poor glycemic control (48.3% patients with HbA1c ≥ 9%). Our study focused on the hospitalized diabetic patients with a large proportion of poor glycemic control.

Our study showed that the CA19-9 level was significantly correlated to HbA1c and total cholesterol. It was shown in Table 2. Other studies found that HbA1c and total cholesterol were independent impact factors for CA199 level [13, 14]. Their results are consistent with our findings. To investigate the mechanism of elevated CA19-9 in diabetic patients, we compared the CRP and fasting C-peptide level between the abnormal CA19-9 group and the normal CA 19 − 9 group. No significant difference was found in the CRP level between the two groups. After expanding the number of cases, we found that there was a significant difference in the fasting C-peptide level between the patients with normal CA19-9 level and those with abnormal CA19-9 level. Poor glycemic control may lead to pancreatic beta cell dysfunction which could be reflected by low fasting C-peptide level [15]. The injury of islet or the pancreas can lead to the increase of CA19-9 [16]. Acute hyperglycemia is a direct trigger of oxidative stress. Oxidative stress is an important mechanism of islet injury [17, 18]. Intracellular cholesterol accumulation leads to islet dysfunction and impaired insulin secretion which provide a new lipotoxicity model [19]. Through in vitro and in vivo experiments, it is found that glycotoxicity and lipotoxicity impair key steps in insulin biosynthesis and release. Hyperglycemia and free fatty acids elicit endoplasmic reticulum and oxidative stress and impair autophagy, causing islet β-cell apoptosis and some glycoprotein components including CA19-9 be released into the blood [19]. Our study found both HbA1c and total cholesterol were the independent contributors to CA19-9. We speculate that the possible mechanism of the elevation of CA19-9 is islet injury caused by glycotoxicity and lipotoxicity. The mechanism of glucose and lipid metabolism disorder and the elevation of CA19-9 needs to be further studied.

In general, our study found that the percentage of subjects exhibiting elevated CA19-9 level is 14% in the HbA1c ≥ 9% diabetic patients, much higher than expected. The underlying mechanism may be related to islet injury caused by glycotoxicity and lipotoxicity.

### Clinical perspectives

Hospitalized patients with diabetes are routinely screened for tumor markers including CA 19 − 9. Our study revealed that the presence of percentage of patients exhibiting elevated CA 19 − 9 level was unexpectedly high in hospitalized diabetic patients with poor hyperglycemia controlled (HbA1c ≥ 9%). A large number of patients with diabetes are found to have abnormal CA19-9 levels. CA19-9 is a tumor marker. Clinicians and patients will worry whether there is a tumor if this indicator is abnormal. They often arrange further examination, including invasive gastroscopy and colonoscopy to exclude cancers. Our findings provide a new interpretation of the abnormal CA19-9 level in diabetic patients with HbA1c ≥ 9%. For the diabetic patients with HbA1c ≥ 9%, abnormal CA19-9 cannot indicate cancer. We suggest reexamine within a few days after normal blood glucose control. It will avoid meaningless examination and unnecessary panic of patients. For the diabetic patients with HbA1c < 9%, further examinations are suggested to exclude cancer soon.

## Data Availability

The datasets during the current study are not publicly available but are available from the corresponding author on reasonable request through email.
